# A case of cervical schwannoma with upper tracheal stenosis followed up as asthma for 4 years

**DOI:** 10.1002/rcr2.1005

**Published:** 2022-07-07

**Authors:** Akira Naomi, Hideyuki Hujiwara, Tatsuhiko Koizumi, Yukari Sakurai, Yasuo Kohashi, Yukiko Yoneda, Yuka Kitamura, Yoshinobu Hattori, Yuji Saitou

**Affiliations:** ^1^ Department of Thoracic Surgery Medical Corporation Kiyosu Respiratory Medical Hospital, Haruhi Respiratory Medical Hospital Achi Japan; ^2^ Department of Respiratory Medicine Medical Corporation Kiyosu Respiratory Medical Hospital, Haruhi Respiratory Medical Hospital Achi Japan

**Keywords:** cervical schwannoma, conservative treatment, hot biopsy forceps, symptomatic asthma, tracheal stenosis

## Abstract

Cases of upper tracheal stenosis due to cervical schwannoma are fairly rare; therefore, no treatment has been determined. In this case, our patient had been treated for asthma for 4 years and was admitted to our hospital because of exacerbation. Computed tomography showed a tracheal stenosis lesion just below the vocal cords, and a biopsy revealed schwannoma. Conservative therapy was preferred rather than tumour resection by surgery. Follow‐up for 5 years showed no changes on imaging. Conservative treatment is considered as an option if the extratracheal tumour does not grow.

## Graphical abstract

We report the diagnosis and conservative treatment for a rare case of cervical schwannoma with severe upper tracheal stenosis.

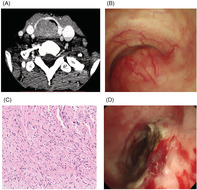

## INTRODUCTION

Tracheal stenosis due to cervical schwannoma is extremely rare, and there is no established treatment method. Because tumours are benign, there is no consensus as to whether surgical resection or conservative treatment should be performed. We report a case of cervical schwannoma with severe upper tracheal stenosis.

## CASE REPORT

**FIGURE 1:**
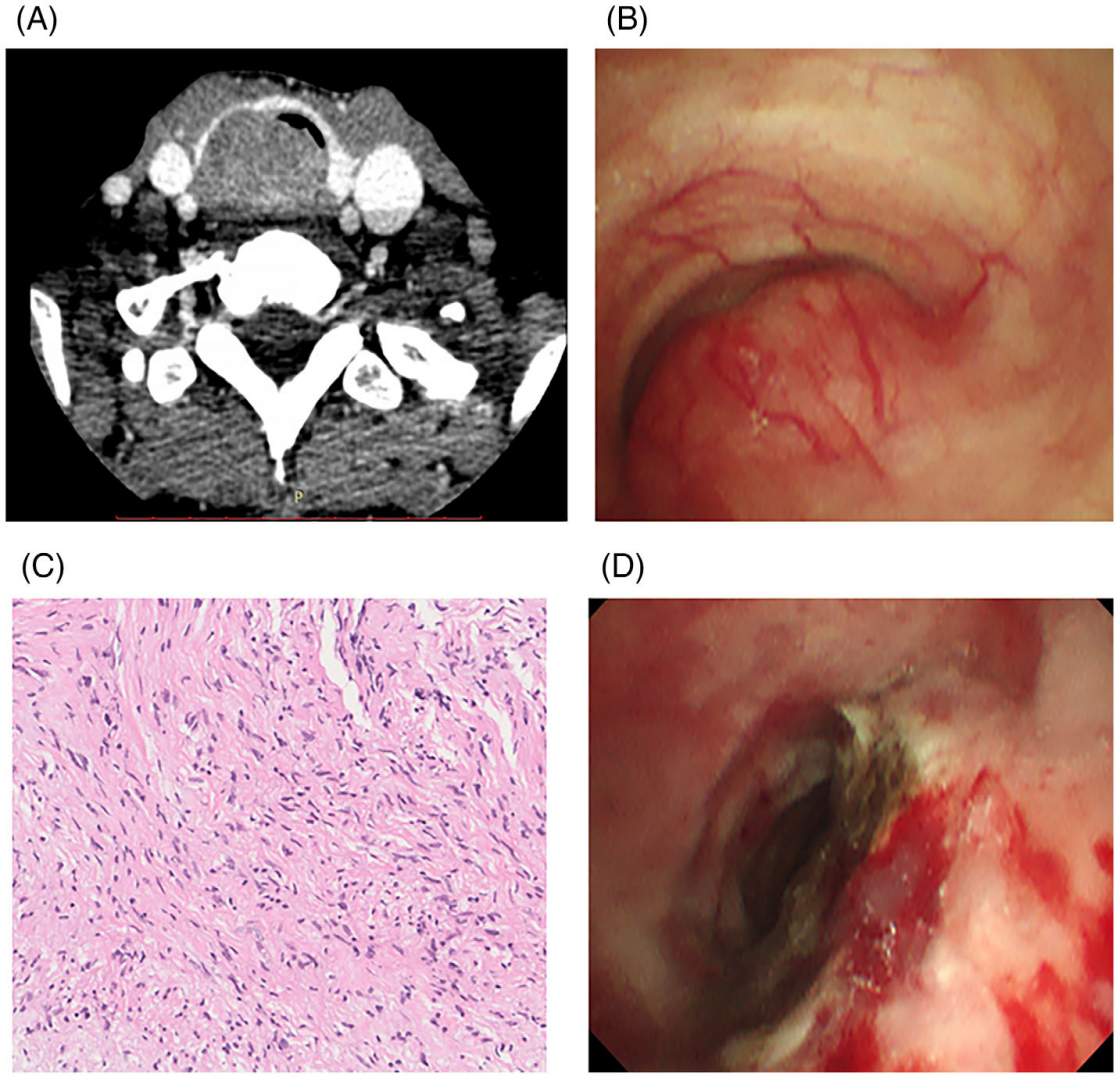
(A) CT showed a solid tumour with a maximum diameter of 37mm in the paratrachea. (B) The tracheal lumen was severely stenotic. (C) The tumour with spindle shaped cells eventually was diagnosed as a low‐grade schwannoma. (D) Intratracheal findings after second tumour removal plus ethanol injection

**FIGURE 2:**
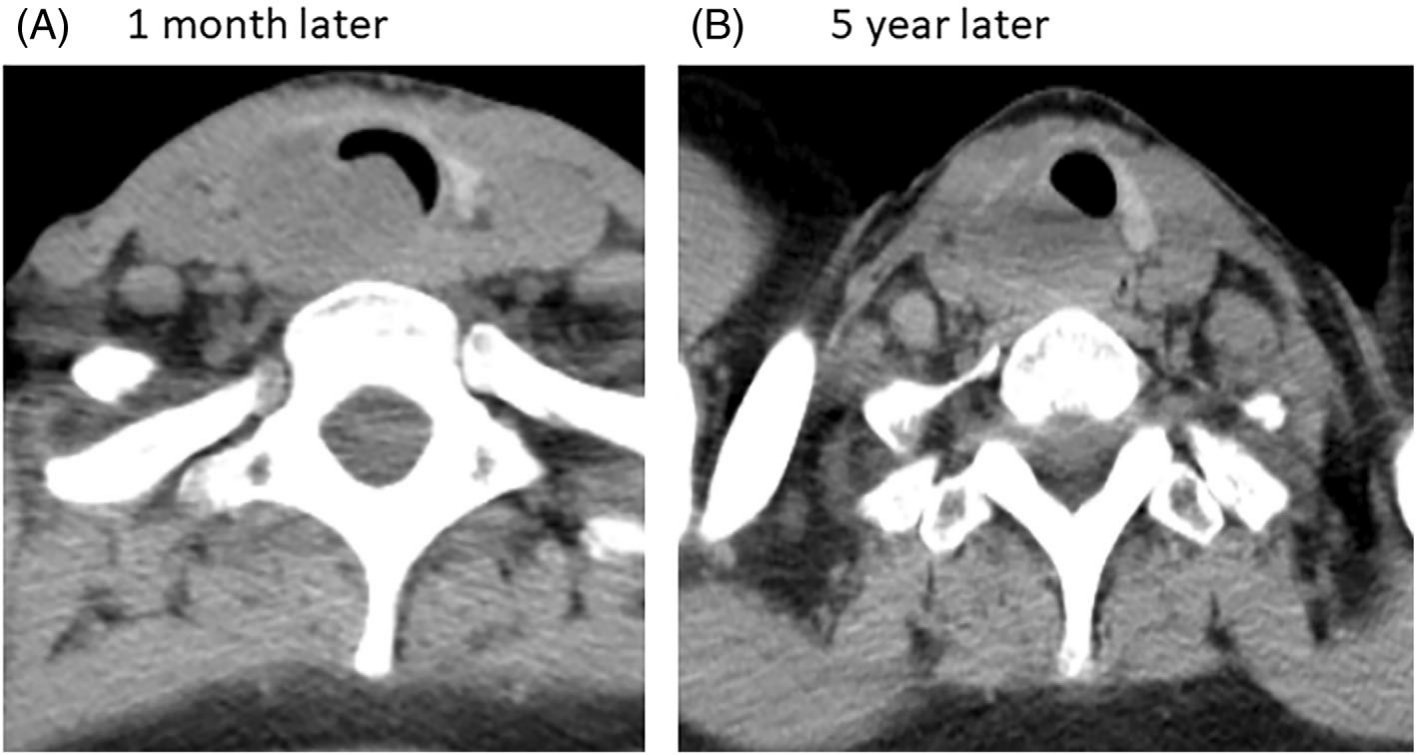
(A) Tumour regrowth in the bronchial lumen was observed 1 month later on relieving intratracheal stenosis. (B) No tendency for tumour growth was observed on computed tomography 5 years later.

A 48‐year‐old woman was diagnosed with bronchial asthma and had been followed up for 4 years. There were no special notes on medical history or family history. At the time of admission, walking was possible with frequent breaks, and her level of dyspnea was considered to be IV degree modified British Medical Research Council (mMRC) according to the Fletcher, Hugh–Jones classification. There was a mildly palpable swollen tumour on the right side of the trachea and no other abnormal findings were found in blood, biochemical findings or tumour markers. Cervical computed tomography (CT) showed a solid tumour with a maximum diameter of 37 mm in the paratrachea which excluded the surrounding tissue (Figure 1A). The tracheal lumen was severely stenotic just below the cricoid cartilage (Figure 1B). A tumour biopsy was attempted with a bronchoscope, but no histopathology was obtained; therefore, a percutaneous paratracheal tumour biopsy was performed. The tumour consisted of spindle‐shaped cells, accompanied by collagen fibres, and no mitotic figures or atypia were observed; immunostaining revealed CD34 (−), S100 (+) and β‐catenin (−), leading to the diagnosis of low‐grade schwannoma. Regarding the treatment policy, we first proposed surgery (partial thyroid cartilage resection plus first to third rings of the trachea plus extra‐cervical tumour resection) to the patient, but she did not agree with it and did not wish to have surgery. Therefore, as a symptomatic treatment, the portion of the tumour causing endoluminal stenosis of the tracheal lumen was removed using hot biopsy forceps under general anaesthesia. Approximately 1 ml of ethanol was injected into the residual intratracheal tumour at five locations. Immediately after tumour removal plus ethanol injection, the ventilator was withdrawn and her wheezing or respiratory distress (present before admission) improved. After about 1 month of rehabilitation, she was discharged on foot. However, approximately 1 month after discharge (Figure 2A), tumour regrowth in the bronchial lumen was observed, and the tumour removal with hot biopsy forceps plus 1 ml of ethanol injection was performed. For the next 5 years, no tendency for tumour growth was observed on CT (Figure 2B).

## DISCUSSION

The number of cases of cervical schwannoma is relatively small, and there are no reports of surgery for upper tracheal stenosis due to cervical schwannoma. Therefore, it can be treated through multiple modalities such as primary tracheal resection or endoscopic treatment, cryotherapy, endoscopic excision and microdebridement.^1^ Hamouri et al.^2^ advise that the choice of treatment should be made by the clinical presentation of the tumour (pedunculated vs. sessile), the risk of tracheal resection and the presence or absence of an extratracheal component. For this case, regarding the treatment of cervical schwannoma, although we first proposed surgery, our patient desired symptomatic treatment mainly for the relief of airway stenosis from the viewpoint of complications and cosmetology. Although rare, there are cases of malignant transformation from schwannoma,^3^, ^4^, ^5^ and if the paratracheal tumour shows a rapid growth tendency, it is necessary to persuade the patient to reconsider surgery. Bronchoscopic ethanol infusion therapy was first reported by Fujisawa et al.^6^ in 1986 for malignant tumours in the central airways. Ethanol injected into the tumour (1) fixes the tumour tissue and shrinks the tumour itself, and (2) blocks the intravascular blood flow on the surface of the tumour, leading to necrosis of the tumour. Currently, it is used for malignant tumours such as haemangiomas and pancreas, cysts, lymphangioma, carcinoids and so on in addition to schwannoma,^7^, ^8^, ^9^ and in some cases, multiple injections of ethanol are needed. The main complications have been reported to be drunkenness, pain, fever, transient hoarseness and cough,^8^, ^9^ but no serious complications have been reported and it is relatively simple and inexpensive. Therefore, if the growth is gradual, ethanol injection may be considered for extratracheal tumours, and symptomatic treatment may be performed for intratracheal tumours by tumour removal plus ethanol injection in cases where conservative treatment is considered an option.

## AUTHOR CONTRIBUTION

Akira Naomi is the primary author of the manuscript, and drafted and revised the paper. Hideyuki Hujiwara, Tatsuhiko Koizumi, Yukari Sakurai, Yasuo Kohashi, Yukiko Yoneda, Yuka Kitamura, Yoshinobu Hattori and Yuji Saitou are the attending doctors who treated the patient on admission. Yuka Kitamura diagnosed the patient pathologically. All authors read and approved the final manuscript.

## CONFLICT OF INTEREST

None declared.

## ETHICS STATEMENT

The authors declare that appropriate written informed consent was obtained for the publication of the manuscript and accompanying images.

## Data Availability

Data sharing is not applicable to this article as no new data were created or analysed in this study.
